# Ethnic disparities in psychotic experiences explained by area-level syndemic effects

**DOI:** 10.1192/bjp.2019.203

**Published:** 2020-10

**Authors:** Jeremy Coid, Rafael Gonzalez Rodriguez, Constantinos Kallis, Yamin Zhang, Kamaldeep Bhui, Bianca De Stavola, Paul Bebbington, Simone Ullrich

**Affiliations:** 1 Professor of Epidemiology in Psychiatry, Mental Health Center and Psychiatric Laboratory, State Key Laboratory of Biotherapy, West China Hospital of Sichuan University, China; and Wolfson Institute of Preventive Medicine, Queen Mary University of London, UK; 2 Post-doctoral Researcher, Wolfson Institute of Preventive Medicine, Queen Mary University of London, UK; 3 Senior Lecturer in Medical Statistics, Wolfson Institute of Preventive Medicine, Queen Mary University of London, UK; 4 Post-doctoral Researcher, Mental Health Center and Psychiatric Laboratory, State Key Laboratory of Biotherapy, West China Hospital of Sichuan University, China; 5 Professor of Psychiatry, Wolfson Institute of Preventive Medicine, Queen Mary University of London, UK; 6 Professor of Medical Statistics, Institute of Child Health, University College London, UK; 7 Professor of Psychiatry, Department of Mental Health Sciences, University College London, UK; 8 Lecturer in Forensic Mental Health, Wolfson Institute of Preventive Medicine, Queen Mary University of London, UK

**Keywords:** Psychotic experiences, syndemic, ethnic/racial disparities, area-level effects, multiple morbidity

## Abstract

**Background:**

Ethnic inequalities in health outcomes are often explained by socioeconomic status and concentrated poverty. However, ethnic disparities in psychotic experiences are not completely attenuated by these factors.

**Aims:**

We investigated whether disparities are better explained by interactions between individual risk factors and place-based clustering of disadvantage, termed a syndemic.

**Method:**

We performed a cross-sectional survey of 3750 UK men, aged 18–34 years, oversampling Black and minority ethnic (BME) men nationally, together with men residing in London Borough of Hackney. Participants completed questionnaires covering psychiatric symptoms, substance misuse, crime and violence, and risky sexual health behaviours. We included five psychotic experiences and a categorical measure of psychosis based on the Psychosis Screening Questionnaire.

**Results:**

At national level, more Black men reported psychotic experiences but disparities disappeared following statistical adjustment for social position. However, large disparities for psychotic experiences in Hackney were not attenuated by adjustment for social factors in Black men (adjusted odds ratio, 3.24; 95% CI 2.14–4.91; *P* < 0.002), but were for South Asian men. A syndemic model of joint effects, adducing a four-component latent variable (psychotic experiences and anxiety, substance dependence, high-risk sexual behaviour and violence and criminality) showed synergy between components and explained persistent disparities in psychotic experiences. A further interaction confirmed area-level effects (Black ethnicity × Hackney residence, 0.834; *P* < 0.001).

**Conclusions:**

Syndemic effects result in higher rates of non-affective psychosis among BME persons in certain inner-urban settings. Further research should investigate how syndemics raise levels of psychotic experiences and related health conditions in Black men in specific places with multiple deprivations.

Incidence and prevalence of psychotic phenomena are highest among persons of Black African and Caribbean origin in the UK^1^ and among African Americans in the USA.^2,3^ To achieve statistical power, incidence studies of ethnic disparities are usually carried out in urban areas where Black and minority ethnic (BME) persons are concentrated and could therefore be influenced by area effects. The highest recorded UK incidence rates were in south and east inner-urban London, areas with exceptional socioeconomic deprivation and ethnic density.^4,5^ Prevalence studies have sampled more widely and report similar results. Because psychotic experiences are relatively common in the general population and on a continuum with psychotic symptoms presenting to clinical services,^6^ measuring psychotic experiences in non-clinical samples is important in disentangling individual-level risk factors, such as ethnicity and socioeconomic influences, from population-level area effects.

There are many proposed explanations for ethnic disparities in psychosis. These include migration, racism and discrimination, ethnic density, access to health services, use of cannabis, biological susceptibility, poorer social support and social disadvantage over the life course.^7,8^ Variations have been demonstrated for a range of other health disparities, but these are largely attenuated following statistical adjustments for socioeconomic factors and neighbourhood characteristics.^9,10^ It is then assumed that socioeconomic factors are responsible. However, in the case of psychotic experiences, disparities persist following adjustment for socioeconomic status,^2^ but less is known about the influence of place and area or neighbourhood characteristics.

An alternative and previously untested hypothesis for observed disparities is that area-level effects operate differentially on BME persons in the form of a syndemic, restricted to certain geographical locations where BME persons are concentrated. These are often deprived inner-city areas. It may be that the findings of disparities are a function of place as much as of individual-level risk factors.

## Syndemic theory

A syndemic is defined by Singer *et al*^11^ as an aggregation of two or more diseases or other health conditions in a population in which there is some level of deleterious biological or behaviour interface that exacerbates the negative health effects of any or all of the diseases involved. Syndemics involve adverse interaction between diseases and health conditions of all types, including psychiatric morbidity and behavioural conditions, and are most likely to emerge under conditions of health inequality caused by poverty, stigmatisation, stress or structural violence.^11^ These factors show synergy, are mutually causative, and studies need to reconsider how to assess causality. The cumulative effect of experiencing these co-occurring problems is therefore greater than experiencing each constituent problem in isolation.^11–13^

Diseases co-occur in particular temporal or geographical contexts because of harmful social conditions (disease concentration) and can interact at the level of populations and individuals, with mutually enhancing deleterious consequences (disease interaction).^11–13^ The first syndemic described consisted of violence, substance misuse and AIDS in poor, minority inner-city populations in the USA, facing multiple political, economic and social challenges of unemployment, substandard housing or homelessness, poor nutrition, disrupted family and social relationships, population displacement and little or no access to healthcare.^14^ Syndemic theory would imply that BME persons living in areas not subject to syndemic effects will not show substantial disparities from the majority White population, and such disparities as emerge will not be attenuated by adjustment for socioeconomic deprivation.

To test this hypothesis, we conducted surveys of young adult men from a representative sample of the UK population and a sample from an area characterised by ethnic and racial diversity with severe socioeconomic deprivation. Our aims were to (a) compare ethnic disparities in the prevalence of psychotic experiences in the general population of the UK with those from a specific deprived area of inner-urban London (BME males compared with the majority White male population); (b) compare BME with White men on a range of mental health and high-risk behaviours; (c) when focussing on psychotic experiences as outcome, investigate whether other health conditions had synergistic effects on their prevalence; and (d) investigate the potential synergistic effects between ethnicity and location on the prevalence of a syndemic comprising four health conditions (including psychotic experiences)

## Method

### Study participants

The Second Men's Modern Lifestyles Survey was carried out in 2011, using random location sampling, an advanced form of quota sampling shown to reduce potential biases introduced when interviewers are able to choose locations to sample from. Individual sampling units (census areas of 150 households) were randomly selected within British regions in proportion to population. The main survey derived a representative sample of young men aged 18–34 years from England, Scotland and Wales. There were two boost surveys: young BME men selected from output areas with a minimum of 5% BME inhabitants, and output areas in the London Borough of Hackney. Hackney was selected for comparison with the general population because of its ethnic diversity and exceptionally high levels of recorded health and social problems. Identical sampling principles were used for each survey type.

A self-administered questionnaire piloted in a previous survey was adapted. Informed consent was obtained from survey respondents who completed pencil and paper questionnaire in privacy and were paid £5 for participation (see Supplementary material available at https://doi.org/10.1192/bjp.2019.203). The study was approved by Queen Mary University London Ethics Committee.

The unweighted sample included 3725 men and the weighted sample included 3750 men, all aged 18–34 years; 1999 (53.3%) were from the representative survey, 991 (26.4%) were from the BME sample and 760 (20.3%) were from Hackney. Because most BME persons in the UK are of Black African or Caribbean origin or from the Indian subcontinent (Indian, Pakistani or Bangladeshi – referred to subsequently as South Asian), we excluded other, smaller BME subgroups from analyses.

### Health-related and other measures

We evaluated 19 health-related measures from 4 different domains: sexual health/risks, defined as ≥10 sexual partners in the past year, contraceptive use rare/never, sex with prostitutes, anal sex, sex with men, forced sex on partners or sexually transmitted infection; substance dependence, defined as alcohol or drug dependence; psychiatric morbidity, defined as anxiety disorder, depressive disorder or psychosis; and violence and criminality, defined as repeated assaults/fights, intimate partner violence, fear of violent victimisation, weapon carrying, gang membership, friends encouraging criminal activity, or imprisonment.

The Psychosis Screening Questionnaire (PSQ)^15^ covers hypomania, thought interference, paranoid ideation, strange experiences and auditory and visual hallucinations in the past year. A positive screening for psychosis was recorded if three or more criteria were met, as in previous surveys.

The Hospital Anxiety and Depression Scale^16^ defined anxiety and depression based on scores ≥11 in the past week. Scores ≥20 on the Alcohol Use Disorders Identification Test^17^ and scores ≥25 on the Drug Use Identification Test^18^ were used to identify alcohol or drug dependence, respectively.

Participants were asked if they had ever seen a psychologist or psychiatrist or had ever been admitted to a psychiatric hospital.

### Statistical analysis

Associations between demography and ethnicity were established through logistic regression analyses. Adjusted models were fitted to study relationships between ethnicity and each health outcome, allowing for differential effects by survey. BME subgroups were contrasted with White men in the main survey. Adjustments were made for age, single status, non-UK birthplace and Index of Multiple Deprivation Rank.^19^ We also compared prevalences of health outcomes among ethnic minority groups in the main and Hackney surveys.

To validate the structure of the health domains and derive domain factor scores, we performed confirmatory factor analysis (CFA). A weighted least square mean- and variance-adjusted estimator was used to account for bias in estimates owing to non-linearity of binary indicators. For identification of the CFA model, item variances were allowed to be estimated freely. The model was standardised by fixing factor variances at 1. Model fit was assessed by root-mean-square error of approximation (RMSEA), the comparative fit index (CFI) and the Tucker–Lewis index (TLI). Values of ≤0.06 for RMSEA, and ≥0.95 for CFI and TLI, indicate very good model fit.^20^ The CFA model showed strong correlations among four health dimensions. To investigate potential syndemic effects, the model was extended to allow for a higher-order latent variable representing a general syndemic dimension, modelled in terms of ethnicity, survey type and their interaction (second-order factor model).

Having used CFA to identify substance misuse, sexual health and violence and crime factors, factor scores derived from this CFA model were then used to examine associations with indicators of psychiatric morbidity (i.e. three or more PSQ criteria and anxiety disorder; prevalence 15%), and their possible interactions. To avoid issues related to collinearity, we examined associations/interactions using two factors at a time (i.e. pairwise): sexual health with substance misuse, and sexual health with violence and crime.

Appropriate non-response weights were used throughout. We used robust s.e. to account for correlations within survey areas owing to clustering within postcodes. A significance level of 0.05 was adopted throughout.

Statistical analyses were performed using Stata version 13 for Windows and Mplus version 7.1 for Windows (Muthén LK and Muthén BO, Los Angeles, USA; see https://www.statmodel.com/).^21^

## Results

### Disparities in psychotic experiences

BME and White men in Hackney differed in sociodemographic characteristics from White men in the general British population (Table 1). Black and South Asian men from the national BME sample were more likely to be born outside the UK and live in areas characterised by multiple deprivation; South Asian men were more likely to be single. In Hackney, White men were more likely to be born outside the UK and fewer were of lower social class and unemployed; Black and South Asian men were more likely to be born outside the UK and fewer were unemployed.

**Table 1:** Sample characteristics according to participant's ethnicity (*N* = 3750)

	Main survey, *N* = 2990 (79.7%), *n* (%)			Hackney survey, *N* = 760 (20.3%), *n* (%)		
	White (reference)	Black	Asian	White	Black	Asian
All participants	1795 (60.1)	492 (16.5)	703 (23.5)	270 (35.5)	259 (34.1)	231 (30.4)
Age, years						
18–24	669 (37.3)	214 (43.6)	273 (38.9)	89 (33.1)	95 (36.9)	75 (32.6)
25–34	1126 (62.7)	278 (56.4)	429 (61.1)	181 (66.9)	163 (63.1)	156 (67.4)
Single	1045 (58.5)	326 (68.3)	450 (66.6)*	177 (65.9)***	143 (55.8)	136 (59.2)
Non-UK born	93 (5.2)	103 (21.9)***	268 (31.5)***	42 (16.1)***	39 (16.0)***	92 (42.7)***
Social class						
I and II^a^	255 (16.9)	48 (13.1)	104 (19.4)	59 (25.4)	40 (19.5)	50 (26.4)
III	565 (37.6)	131 (36.2)	152 (28.4)	75 (32.3)*	71 (34.4)	69 (36.8)
IV and V	388 (25.8)	109 (30.2)	187 (35.0)	58 (25.2)*	63 (30.6)	57 (30.3)*
Unemployed	297 (19.7)	74 (20.5)	93 (17.3)	40 (17.1)***	32 (15.5)**	12 (6.5)***
IMDR (mean)	12 370.3	7897.9***	8010.0***	4656.2***	4658.8***	4437.3***

All data are weighted frequencies and percentages (row %). Adjusted for the other sociodemographic characteristics and Index of Multiple Deprivation Rank (IMDR).

a. Association test based on multinomial logistic regression with social classes I and II as base level. Social class was assessed using the Standard Occupational Classification 1991.^22^

*P < 0.05, **P < 0.01, ***P < 0.001, in reference to White men in the main survey.

Before adjustment, Black men in the national survey showed a higher mean number of psychotic experiences (particularly thought insertion) than their White counterparts (Supplementary Table 1). After adjustment, no differences were found among Black and Asian men for number of psychotic experiences, one or more psychotic experiences, a categorical classification of psychosis (using PSQ threshold of three or more endorsed items), associated healthcare use including consulting a psychologist/psychiatrist, hospital admission or any of five individual symptoms (Table 2).

**Table 2:** Adjusted effects on psychotic experiences according to BME group and survey

	White			Black				Asian			
Psychosis measures	Main^a^	Hackney		Main		Hackney		Main		Hackney	
	*n* (%)	*n* (%)	AOR (95% CI)	*n* (%)	AOR (95% CI)	*n* (%)	AOR (95% CI)	*n* (%)	AOR (95% CI)	*n* (%)	AOR (95% CI)
No. of symptoms (mean, s.d.)	0.21 (0.62)	0.34 (0.80)	1.68 (1.13–2.49)*	0.38 (0.82)	1.69 (1.11–2.56)*	0.70 (1.21)	3.47 (2.03–5.91)***	0.17 (0.54)	0.78 (0.46–1.34)	0.36 (0.94)	1.43 (0.87–2.36)
PSQ 1+	230 (13.4)	54 (20.7)	1.70 (1.14–2.54)*	118 (24.6)	1.77 (1.14–2.75)*	84 (33.4)	3.14 (1.92–5.15)***	81 (12.1)	0.78 (0.45–1.36)	39 (17.4)	1.37 (0.84–2.23)
PSQ 3+	33 (1.9)	8 (3.1)	1.59 (0.67–3.75)	13 (2.7)	0.55 (0.19–1.57)	27 (10.6)	6.01 (2.84–12.68)***	9 (1.3)	0.59 (0.24–1.43)	10 (4.6)	2.33 (0.98–5.54)
Hypomania	40 (2.3)	11 (4.3)	1.70 (0.80–3.62)	11 (2.3)	0.44 (0.10–1.91)	28 (11.2)	4.37 (2.29–8.33)***	12 (1.8)	0.48 (0.20–1.13)	18 (7.8)	3.64 (1.91–6.92)***
Thought insertion	31 (1.8)	12 (4.6)	3.16 (1.61–6.21)**	29 (6.0)	2.62 (1.23–5.60)**	28 (11.1)	6.53 (3.04–14.04)***	16 (2.4)	1.48 (0.58–3.82)	15 (6.8)	4.66 (2.16–10.07)***
Paranoid ideation	77 (4.5)	10 (4.0)	0.99 (0.51–1.89)	31 (6.5)	1.24 (0.64–2.42)	43 (17.4)	4.77 (2.53–9.01)***	16 (2.4)	0.38 (0.19–0.74)*	11 (4.7)	0.94 (0.41–2.15)
Strange experiences	99 (5.8)	21 (8.2)	1.51 (0.86–2.67)	47 (9.8)	1.22 (0.65–2.29)	42 (16.9)	3.99 (2.23–7.17)***	26 (3.8)	0.61 (0.34–1.08)	16 (6.9)	1.00 (0.47–2.12)
Hallucinations	39 (2.3)	9 (3.5)	1.68 (0.76–3.70)	14 (2.9)	1.16 (0.44–3.04)	16 (6.3)	2.55 (1.18–5.52)	6 (0.9)	0.29 (0.09–0.98)	8 (3.6)	1.77 (0.54–5.78)
Psychiatrist/psychologist and PSQ 3+	5 (0.3)	3 (1.2)	2.36 (0.42–13.40)	1 (0.2)	0.66 (0.07–6.64)	4 (1.7)	6.21 (1.61–23.93)*	1 (0.1)	0.47 (0.08–2.74)	1 (0.5)	1.72 (0.22–13.52)
Admission to psychiatric hospital and PSQ 3+	8 (0.5)	2 (0.9)	1.44 (0.27–7.60)	0 (0.0)	–^b^	8 (3.5)	5.73 (1.83–17.96)*	1 (0.2)	0.46 (0.11–1.86)	5 (2.6)	4.88 (1.76–13.52)*

All data are weighted frequencies, percentages and estimates (AOR, 95% CI). Adjusted for age, being single, non-UK born, social class and IMDR. With Bonferroni correction (based on five estimates for each outcome).

BME, Black and minority ethnic; AOR, adjusted odds ratio; PSQ, Psychosis Screening Questionnaire; IMDR, Index of Multiple Deprivation Rank.

a. In reference to White men in the main survey.

b. Estimate not obtained because of data sparseness.

*P < 0.01, **P < 0.002, ***P < 0.0002.

However, Black men in Hackney showed multiple differences on all variables, except hallucinations. They were also more likely to have been admitted to a psychiatric hospital. South Asian men in Hackney showed similar trends, although only symptoms of hypomania, thought insertion and hospital admission differed significantly from White men. White men in Hackney were more likely to report thought insertion than Black men.

### Disparities in health measures

Supplementary Figure 1 shows distributions of each health measure among ethnic groups compared with White men in the main national survey. Following adjustments, Black men in the main survey were significantly less likely to report anal sex or sex with men. Only ≥10 sexual partners in the past year was more frequently reported (Table 3). South Asian men in the main national survey were significantly less likely to report sex with prostitutes, anal sex, repeated violence and friends encouraging criminal behaviour. Anxiety disorder was more frequent.

**Table 3:** Adjusted effects on health measures according to BME group and survey

	White		Black		Asian	
	Main	Hackney	Main	Hackney	Main	Hackney
Health outcomes by domain		AOR (95% CI)	AOR (95% CI)	AOR (95% CI)	AOR (95% CI)	AOR (95% CI)
Substance misuse						
Alcohol dependence	Reference	4.54 (3.00–6.87)***	1.98 (1.00–3.91)	6.97 (4.06–11.96)***	1.23 (0.59–2.57)	2.89 (1.64–5.08)**
Drug dependence	Reference	0.34 (0.08–1.41)	0.30 (0.10–0.88)	12.58 (6.26–25.25)***	0.59 (0.15–2.41)	2.13 (0.84–5.38)
Mental health						
Psychosis (PSQ 3+)	Reference	1.59 (0.67–3.75)	0.55 (0.19–1.57)	6.01 (2.84–12.68)***	0.59 (0.24–1.43)	2.33 (0.98–5.54)
Anxiety disorder	Reference	2.78 (1.90–4.06)***	1.20 (0.69–2.06)	4.92 (2.86–8.49)***	2.61 (1.71–4.00)***	3.85 (2.57–5.76)***
Depressive disorder	Reference	3.89 (2.46–6.14)***	1.30 (0.68–2.50)	4.67 (2.86–7.64)***	1.18 (0.70–2.00)	5.66 (3.42–9.35)***
Sexual health						
STD	Reference	1.01 (0.61–1.69)	1.11 (0.64–1.93)	2.52 (1.35–4.71)*	0.35 (0.14–0.87)	0.91 (0.49–1.70)
Paid sex worker (ever)	Reference	1.13 (0.62–2.04)	1.00 (0.59–1.70)	3.71 (2.18–6.29)***	0.35 (0.16–0.77)*	2.13 (1.28–3.53)*
Anal sex	Reference	1.17 (0.79–1.73)	0.39 (0.23–0.66)**	1.78 (1.13–2.80)**	0.27 (0.16–0.47)***	0.43 (0.26–0.73)**
Coercive sex	Reference	1.32 (0.72–2.41)	0.86 (0.37–1.97)	3.60 (2.04–6.37)***	1.33 (0.58–3.05)	2.70 (1.30–5.62)*
≥10 sexual partners (past year)	Reference	4.96 (3.01–8.15)***	4.23 (2.40–7.46)***	7.67 (4.64–12.67)***	0.66 (0.24–1.83)	6.18 (3.09–12.38)***
Rare contraceptive use	Reference	1.24 (0.86–1.78)	1.12 (0.77–1.62)	2.17 (1.41–3.33)**	1.12 (0.81–1.55)	1.96 (1.26–3.06)*
Sexually active MSM	Reference	1.17 (0.57–2.40)	0.19 (0.07–0.53)**	3.67 (1.71–7.86)**	0.68 (0.24–1.91)	1.63 (0.79–3.36)
Violence and criminality						
Repeated violence	Reference	0.52 (0.17–1.54)	0.42 (0.20–0.90)	3.80 (2.08–6.95)***	0.16 (0.07–0.38)***	0.84 (0.37–1.91)
IPV	Reference	0.33 (0.10–1.06)	0.53 (0.23–1.23)	7.03 (3.85–12.84)***	0.63 (0.24–1.62)	1.97 (0.90–4.31)
Fear of violent victimisation	Reference	0.99 (0.57–1.72)	1.30 (0.76–2.20)	3.93 (2.31–6.66)***	1.64 (1.12–2.41)**	1.70 (1.03–2.82)
Carried a weapon	Reference	0.83 (0.47–1.50)	0.67 (0.34–1.29)	4.74 (2.44–9.19)***	0.80 (0.35–1.85)	0.93 (0.45–1.89)
Gang membership	Reference	0 cell	0.78 (0.20–2.94)	42.36 (15.81–113.52)***	1.35 (0.48–3.75)	6.55 (2.00–21.46)**
Peers encourage crime	Reference	0.74 (0.40–1.35)	0.68 (0.42–1.12)	2.53 (1.44–4.45)**	0.20 (0.11–0.37)***	1.96 (1.12–3.44)*
Ever in prison	Reference	0.57 (0.23–1.40)	0.77 (0.39–1.53)	2.54 (1.19–5.41)**	0.13 (0.02–0.81)***	1.00 (1.00–1.00)^a^

All data are weighted estimates (AOR, 95% CI). Adjusted for age, being single, non-UK born, social class and IMDR. With Bonferroni correction (based on five estimates for each outcome).

BME, Black and minority ethnic; AOR, adjusted odds ratio; PSQ, Psychosis Screening Questionnaire; STD, sexually transmitted diseases; MSM, men who have sex with men; IPV, intimate partner violence; IMDR, Index of Multiple Deprivation Rank.

a. Did not meet complete separation assumption for binary logistic regression.

*P < 0.01, **P < 0.002, ***P < 0.0002.

Compared with White men in the main national survey, White men in Hackney were more likely to report ≥10 sexual partners in the past year, alcohol dependence, anxiety disorder and depressive disorder (Table 3). Black men in Hackney were more likely to report a wide range of adverse health measures, including sexually transmitted infections, sex with prostitutes, coercive sex, ≥10 sexual partners, rarely/never using contraceptives, sex with men, alcohol dependence, drug dependence, anxiety disorder, depressive disorder, psychosis, repetitive violence, intimate partner violence, fear of violent victimisation, weapon carrying, gang membership, friends encouraging criminal activity, and imprisonment than White men in the main survey. South Asian men in Hackney were less likely to report anal sex but more likely to report sex with prostitutes, coercive sexual behaviour, ≥10 partners in the past year, rarely/never using contraceptives, alcohol dependence, anxiety disorder, depressive disorder and gang membership than White men in the main survey.

### Syndemic model

We developed a syndemic model, using CFA to include measures of psychiatric morbidity, substance dependence, sexual health and violence and crime. The first step involved identifying first order syndemic factors by domain. The gang membership variable was highly skewed (no White respondents reported gang membership in Hackney) and excluded owing to poor convergence. All items loaded highly except depression and never/rare use of contraceptives. However, these were retained on the basis of clinical relevance. Model fit indices were excellent (RMSEA = 0.025, 95% CI 0.022–0.027, CFI = 0.960, TLI = 0.952).

Because first-order syndemic factors were highly correlated, we followed standard SEM modelling practice by developing a second-order CFA, thereby generating a general syndemic factor that linked first-order health factor domains. Supplementary Figure 2 confirms that a second-order syndemic factor should be included in the model (fit indices: RMSEA = 0.025, 95% CI 0.022–0.027, CFI = 0.959, TLI = 0.951).

Table 4 shows evidence of syndemic effect (i.e. positive interaction) between high-risk sexual behaviour and violence/criminality factors in the association with psychotic experiences and anxiety. Similarly, there was evidence of a syndemic effect between the substance misuse and sexual health on the psychotic experiences and anxiety outcome. In the presence of both pairs of factors (substance misuse × sexual health; sexual health × violence and criminality), their combined associations with psychotic experiences and anxiety were significantly increased.

**Table 4:** Associations and synergy between substance misuse (SM), violence/crime (VC) and sexual health (SH) factors with psychotic experiences/anxiety (PA) outcome^a^ (*N* = 3,750)

	Direct associations with PA	Synergy between factors in associations with PA
*Factor*	OR (95% CI)	OR (95% CI)
SM	3.33 (2.94–3.77)**	
VC	3.03 (2.69–3.41)**	
SH	2.90 (2.57–3.27)**	
*Interactions*		
SM by VC^b^		−
SM by SH		1.17 (1.00–1.36)*
SH by VC		1.17 (1.01–1.36)*

OR, odds ratio.

a. PSQ 3+/anxiety.

b. Factors were highly correlated.

*P < 0.05, **P < 0.001.

Supplementary Table 2 compares BME groups defined by location for the general syndemic factor. In the main national survey, there were no significant differences in factor scores between Black and White men. However, scores for South Asian men were significantly lower (*P* < 0.001) than the White group.

Scores for White, Black and South Asian subgroups in Hackney were significantly higher compared with White men in the main national survey (*P* < 0.001). We estimated the effect of location (i.e. moderation) on ethnic differences in the latent syndemic score. Supplementary Table 2 shows evidence of significant moderation by location on BME group differences from White men. More specifically, being in Hackney significantly increased the latent syndemic score by 0.834 (*P* < 0.001) among Black men, and by 0.376 (*P* < 0.05) among South Asian men.

## Discussion

Our findings are new and describe a syndemic of four health conditions: psychotic experiences and anxiety, substance dependence, high-risk sexual behaviour and crime and violence. After selecting psychotic experiences as outcome of interest, we demonstrated synergy between other components on prevalence of men reporting three or more psychotic experiences and severe anxiety. We additionally showed important area-level effects on psychotic experiences. Most significantly, BME in the national population did not show disparities in psychotic experiences observed in previous population studies after adjusting for both social status and neighbourhood effects. This corresponds to US studies of other health outcomes.^9,10^ In addition, they did not report more substance dependence, high-risk sexual behaviour, crime/violence or poorer mental health, with the exception being anxiety disorder among South Asian men. In marked contrast, Black men in Hackney showed higher number of psychotic experiences, more had putative diagnoses of psychosis and all had higher levels of all psychotic experiences, except hallucinations. We also demonstrated an interaction between Black ethnicity and living in Hackney on prevalence of the syndemic (comprising all four health conditions) to indicate location and that Black men were affected most severely by the syndemic.

To the best of our knowledge, the prevalence of Black men in Hackney reporting psychotic experiences was the highest reported among studies using the PSQ. They were also more likely to have consulted a psychiatrist or psychologist and been admitted to a psychiatric hospital. South Asian men were more likely to have experienced thought insertion and hypomania, and to have been admitted to hospital. Anxiety and depressive disorder, alcohol dependence, high-risk sexual behaviour and gang membership were also more prevalent among Black and South Asian men in Hackney, and Black men reported more violence and criminality. White men in Hackney also reported more anxiety and depression, alcohol dependence and more sexual partners than their counterparts in the general population, showing that all ethnic groups are vulnerable to area effects, and that area × ethnicity interactions should be studied in future research.

We identified a four-component syndemic that explained our findings. First, we used an aggregation of four health conditions by factor analysis. Second, we examined the interactions between these health conditions, which included substance dependence, high-risk sexual behaviour and violence and criminality on psychotic experiences and anxiety disorder. Although each component represented different domains and pathways, the clustering of multiple risk factors increased the prevalence of psychotic experiences, and vice versa. These analyses correspond to recommendations that multiplicative effects should be demonstrated if the aim is to confirm synergy between hypothesised components of a syndemic.^12^ Third, we examined an additional interaction analysis showing strong area-level effects, which located the syndemic primarily in Hackney and severely affected Black and, to a lesser extent, young South Asian men.

Although social inequality plays a crucial role in the clustering of syndemic factors,^23^ neither social status nor neighbourhood deprivation attenuated the disparities we observed in Hackney. Furthermore, because BME and White subgroups in Hackney were more likely to be employed than their counterparts in the general population, syndemic effects were not simply related to low socioeconomic status.

### Ethnic disparities and syndemic effects

An alternative explanation for previously recorded disparities in psychosis is that syndemic effects differentially affect BME and other socially segregated and excluded subgroups, but in a relatively small number of inner-urban areas characterised by extreme socioeconomic deprivation. These may have disproportionate effects on recorded levels of clinical psychosis in these areas explained by synergy between psychotic experiences and anxiety and severe substance misuse, but also between health-related sexual behaviours and violent/criminal behaviour. The demographic characteristics of Hackney and its level of health problems are seen only in a very small number of inner-urban UK areas. Our findings suggest that BME persons in these areas are unrepresentative of BME persons in national samples and those located elsewhere in the UK, being subject to an excess of risk factors that remain unknown and require further investigation.

The components of the syndemic we identified largely correspond to risk factors previously identified for psychotic experiences, including alcohol and cannabis misuse,^24^ same-sex sexual behaviour,^25^ anxiety disorder,^26^ traumatic experiences and stress,^27^ low social cohesion and crime victimisation.^28^ Bi-directionality between psychotic experiences and risk factors has been specifically demonstrated in alcohol misuse and anxiety disorders (including post-traumatic stress disorder), as well as for psychotic illness.^29^ However, most studies investigate single risk factors which, although strongly associated with psychosis, are not by themselves sufficient to explain a large excess of either psychotic experiences or clinical psychosis in a population.

### Pathways and mechanisms

Further research is required into specific pathways through which the psychiatric morbidity and health conditions we have described interact both in individuals and populations to allow multiplication of adverse health effects.^11,12^ Being exposed to violence, both as victim and perpetrator in violent neighbourhoods, particularly in the context of gang membership, is strongly associated with the specific association between psychotic symptoms and anxiety.^30^ The pathway of this relationship is believed to be stress-promoted immune system deregulation caused by living in a pervasive atmosphere of fear and the perceived threat of ever-present violence.^11,30^

Sexual health and high-risk sexual behaviour correspond to other components of the syndemic. Sexually transmitted diseases, particularly HIV, are associated with anxiety and depression together with substance misuse^31^ and non-sexual violence.^32^ Furthermore, recent research into addictions and addictive behaviours has identified underlying similarities between sexual behaviours (which have features of impulsivity, compulsivity, negative emotional states and craving) and substance use disorders, suggesting common causes^32^ and possible pathways to other psychiatric and cognitive disorders, constituting a basis for dual-diagnosis disorders.^33^ This interpretation fits the notion that syndemic effects lead to illnesses, that these are a manifestation of individual risks and area effects and that multiple disadvantages are pathways to multiple illnesses, which mutually increase risks.

### Limitations

Our study has several limitations. First, our hypothesis that a multi-component syndemic may explain high rates of psychotic experiences is currently based on results from a single geographical location (Hackney). However, there are a small number of other UK inner-urban areas with similar levels of socioeconomic deprivation together with high levels of ethnic density, in which our findings may be replicated in the future. Furthermore, Hackney was one of three boroughs in east London found to have exceptional incidence rates of psychosis among BME persons a decade earlier.^5^

Although the PSQ assesses only five symptoms, these are central to the diagnosis of psychosis, have good face validity and are likely to be associated with other symptoms in the syndrome of psychosis. It has been the most extensively used instrument to self-report psychotic experiences in UK surveys, with a cut-off of three items shown to have good accuracy for clinical psychosis.^34^

Although we demonstrated synergy between components of the syndemic, these were highly collinear and, in the case of the association between violence/criminality and substance misuse with psychotic experiences and anxiety as outcome, it was not possible to demonstrate an interaction owing to collinearity.

Because White and BME groups in the UK live in closer physical proximity in areas of concentrated poverty than the USA, this may explain why adjustments for socioeconomic deprivation, measured at small area level, did not show the same attenuation between disparities observed in US studies.^9,10^

Our survey was also restricted to young adult men. Previous US studies of syndemics have emphasised the importance of substance misuse, HIV risk behaviour and violent victimisation (substance abuse, violence and AIDS syndemic (SAVA)) among BME women.^35^ However, these have not investigated psychosis. Furthermore, the relationship between violent victimisation reported by these women and high levels of self-reported violent perpetration by men in this study raises the question of whether there is a close association between the two syndemics. This would require further investigation among men in US inner-urban areas where SAVA has been identified in women.

Random location sampling does not provide detailed information on number of young men who declined to participate. However, because the method is based on the National Census, participants were identified and included according to representative strata and their actual frequency in the population. Because young adult men of lower social class are more likely to decline participation in adult household surveys, this method has considerable advantages for investigating health-related behaviours such as violence and criminality, substance misuse and sexual behaviour. The alternative would be to rely on a method requiring statistical weighting to adjust for attrition, which may be particularly high among this subgroup of the population. However, statistical power and effect calculations assume probability sampling and do not technically apply to quota samples like this (see Supplementary material).

### Implications

Our study suggests a new theoretical explanation for ethnic disparities in psychosis observed in UK inner-urban areas.^4,5^ It corresponds to research emphasising social factors in the study of psychosis and urbanicity, indicating effects of social adversity and exclusion in relation to observed geographical variation. Although the study is primarily from the perspective of psychotic experiences as the primary outcome, bi-directionality with anxiety, drug and alcohol misuse, high-risk sexual behaviour and violence and criminality should also be considered, given that each are potential outcomes and equally dependent on each other. The syndemic therefore corresponds to studies from the early 20th century onward, which have reported that these components co-occur and cluster geographically.^36,37^

The prevalence of psychotic experiences originally measured at baseline corresponds to persistence rather than remission of psychotic experiences and later transition to psychotic disorder.^38^ The syndemic we identified could therefore explain both the higher prevalence of psychotic experiences and raised incidence rates of non-affective psychosis among BME persons previously observed in inner-urban areas characterised by concentrated deprivation.^1,4,5^ Our findings also provide a plausible explanation for exceptional levels of demand on clinical and social services in these areas.

Synergy between the four domains of the syndemic may increase the risk of transition to clinical psychosis, representing a significant public mental health problem. This requires further investigation in longitudinal studies. Environmental exposures, particularly stress factors, are believed to interact with genetic risk in modifying biological pathways to psychotic experiences and, with increased stress sensitivity, to schizophrenia. Hyperactivity of the hypothalamic-pituitary-adrenal axis and increased sensitivity to stress promote emergence of psychotic experiences.^38^ Transitory developmental expression of psychotic experiences may persist and progress to clinical impairment depending on the degree of environmental risk the person is additionally exposed to.^6,38^ The syndemic effects we identified would therefore imply exceptional risks, further magnified by synergy between these risk factors. However, because these risks are substantially increased by specific health-related behaviours, interventions are needed to discourage progression from psychotic experiences to clinical psychosis by encouraging reduction of these behaviours in vulnerable populations.

A key finding was that most BME men in the UK function as well as their White counterparts despite being more likely to live in lower income households. Young BME men can therefore clearly overcome adverse factors contributing to mental health disparities. However, syndemic effects have a differential impact. It may thus be difficult for BME men to avoid these adverse outcomes when living in areas of concentrated poverty and ethnic density where high-risk behaviours may be condoned and encouraged within socially isolated and excluded population subgroups. Moreover, such behaviours are partly determined by multiple political, economic and social challenges beyond the control of these populations. Interventions that promote mental health equality by reducing the gap between the most and least deprived have been recommended.^39^ However, our findings suggest that additional interventions will be needed to promote behavioural change and healthier lifestyles to avoid risks from the four domains we identified. Improved accessibility and quality of clinical services in these areas through adequate funding are needed.^39^ However, resources are also required to develop new primary prevention strategies, and these may need to target behavioural change to reduce transition to clinical psychosis. A narrow clinical focus on psychotic experiences alone is unlikely to be effective as each component of the syndemic may require specific interventions to reduce transition to clinical psychosis. Finally, we require a public mental health approach to prevention and control, not only of the syndemic components, but the forces that originally determined and now tie these components together.^11,13^

## Supplementary material

For supplementary material accompanying this paper visit https://doi.org/10.1192/bjp.2019.203. click here to view supplementary material
