# Supplementary material for: Ethnic disparities in psychotic experiences explained by area-level syndemic effects
Source: Br J Psychiatry. 2019 Oct 30;217(4):555–61. doi: 10.1192/bjp.2019.203 (PMC7525103; doi:10.1192/bjp.2019.203)

*Supplementary text: Quota Sampling and the 2011 Young Men's Health Survey - Report from ICM.*

*Individuals were recruited by proportional quota sampling. This is a standard method that entails setting quotas for participants on a range of demographic factors and ensures that the sample interviewed is representative of the population of interest. It is particularly useful when investigating hard-to-reach samples, sensitive subjects such as violence, sexual behaviour, etc, and where it is anticipated that certain sub-sections of the population are less likely to complete interviews or questionnaires (e.g. young, male, lower social class, ethnic minorities, in areas of socioeconomic deprivation).*

*Quota sampling offers an alternative to probability sampling and is often used in market research and national surveys and becomes necessary if there is no listing of all those eligible to be included. It is more efficient as recruitment and sampling can be focused in areas in which the desired population are resident but does require good census data on the characteristics by which the quota are set. This method is preferred if the costs of probability sampling would be prohibitive and where feasibility issues become prohibitive.*

*Information on number of questionnaires returned was not collected because it is not a requirement of the methodology used here. The 2011 Young Men's Health Survey – and previous waves of research – adopted an in-home ‘random location’ with respondent self-completion and interviewer pick-up. Random location is the most common form for social and public policy research surveys as it combines a rigorous methodology with relatively low costs.*

*In this particular survey, we used Output Areas as the principal sampling unit. OAs are the base unit of the Census outputs and are based on groups of postcodes that fit within the boundaries of electoral wards/divisions and parishes. OAs represent the lowest geographical level on which full information can be generated through Census output. Each OA contains approximately 150 households and interviewers were required to achieve a target of 13 interviews (i.e. questionnaire pick-ups) in each OA.*

*A quota sheet was provided for each selected grouped OA, which reflects the actual composition of OA residents according to standard demographic criteria. Interviewers were required to interview a sample profile that exactly matches that of the grouped OA population profile using Census population information. This ensures that the sample is demographically representative at the micro-level, as well as geographically representative.*

*Crucially, interviewers were not required to record the number of questionnaires handed out, meaning it is not possible to record a response rate. This is in contrast to a random probability (pre-selected) survey where interviewers are required to visit randomly selected households and interview randomly selected individuals. With this technique, the response rate is recorded and taken as a measure of quality and robustness of the sample.*

*However, it is possible to work out an indicative response rate to the 2011 Young Men's Health Survey. ICM interviewers are required to leave a minimum of three addresses between calls, as well as include evening and weekend visits so that they maximise the chance of meeting their quotas and to avoid clustering in a single street. As such, it is reasonable to assume that interviewers distributed questionnaires to no more than a quarter of the households in each OA. This being the case, an approximate response rate would be around 35% (i.e. 13 completed surveys per point from 37.5 households visited). Nonetheless, this is an approximation and cannot be regarded as a true response rate.*

*Translation by interviewers was not allowed which meant that to complete questionnaires the participants had to be English speakers.*

*Information on missing data from questionnaires is given in supplementary Table 3.*

*Supplementary Table 1. Unadjusted effects on psychotic experiences according to BME group and survey*

|  | White | | Black | | Asian | |
| --- | --- | --- | --- | --- | --- | --- |
| Psychosis measures | Main | Hackney | Main | Hackney | Main | Hackney |
| No. of symptoms (M, SD) | Ref. | 1.68* (1.18 -2.37) | 2.06** (1.36 – 3.12) | 3.54*** (2.27 – 5.52) | 0.88 (0.58 – 1.32) | 1.42 (0.95 – 2.12) |
| PSQ 1+ | Ref. | 1.68* (1.19 – 2.39) | 2.11** (1.40 – 3.19) | 3.24*** (2.14 – 4.91) | 0.89 (0.59 – 1.35) | 1.36 (0.92 – 2.00) |
| PSQ 3+ | Ref. | 1.65 (0.77 -3.54) | 1.42 (0.58 – 3.53) | 6.07*** (3.22 – 11.44) | 0.66 (0.32 – 1.37) | 2.45 (1.14 – 5.27) |
| Hypomania | Ref. | 1.92 (1.00 – 3.70) | 0.99 (0.36 – 2.74) | 5.38*** (3.20 – 9.06) | 0.78 (0.36 – 1.68) | 3.60*** (1.99 – 6.51) |
| Thought insertion | Ref. | 2.60* (1.38 – 4.87) | 3.38** (1.76 – 6.50) | 6.63*** (3.58 -12.27) | 1.32 (0.61 – 2.89) | 3.89*** (2.08 – 7.27) |
| Paranoid ideation | Ref. | 0.88 (0.48 – 1.62) | 1.50 (0.62 -3.64) | 4.51*** (2.62 – 7.78) | 0.52 (0.31 – 0.90) | 1.04 (0.52 – 2.08) |
| Strange experiences | Ref. | 1.45 (0.90 – 2.35) | 1.77 (0.95 – 3.32) | 3.33*** (2.03 -5.47) | 0.65 (0.41 – 1.03) | 1.21 (0.69 – 2.14) |
| Hallucinations | Ref. | 1.56 (0.79 – 3.12) | 1.29 (0.51 – 3.27) | 2.85** (1.54 – 5.29) | 0.39 (0.17 – 0.91) | 1.61 (0.61 – 4.22) |
| Psychiatrist/psychologist & PSQ 3+ | Ref. | 4.64 (1.13 – 19.00) | 0.67 (0.08 – 5.68) | 6.21* (1.78 – 21.64) | 0.49 (0.09 – 2.50) | 1.77 (0.21 – 15.19) |
| Admission to psychiatric hospital & PSQ 3+ | Ref. | 1.76 (0.37 – 8.31) | -**** | 7.33** (2.54 – 21.12) | 0.44 (0.12 – 1.70) | 5.41* (1.84 – 15.89) |

Note: All weighted estimates (AOR, 95% CI).

With Bonferroni correction (based on 5 estimates for each outcome): *** p < 0.0002; ** p < 0.002; * p < 0.01.

****: estimate not obtained due to data sparseness

| *Supplementary Table 2. Test of effect-modification of ethnicity by survey location on the general syndemic factor* | | | | | | | | |
| --- | --- | --- | --- | --- | --- | --- | --- | --- |
|  | Main | | | Hackney | | | Location by ethnic differences^a^ | |
|  | White | Black | Asian | White | Black | Asian | Black | Asian |
|  |  | coefficient | coefficient | coefficient | coefficient | coefficient | contrast | contrast |
| General *syndemic* factor | Ref. | -0.066 | -0.371^†^ | 0.409^†^ | 1.177^†^ | 0.414^†^ | 0.834^†^ | 0.376^#^ |
| Reference: main survey White  ^a^Location contrasts for ethnic group’s difference in coefficient from White  ^#^P < 0.05, ^¶^P < 0.01, ^†^P < 0.001 | | | | | | | | |

| *Supplementary Table 3. Patterns of missing values on Youth Male Survey sample* | | |
| --- | --- | --- |
|  | Missing frequency | Missing % |
| Survey type | 0 | 0 |
| Ethnicity | 0 | 0 |
| Age | 0 | 0 |
| Being single | 0 | 0 |
| Non-UK born | 73 | 1.96 |
| Social class / Unemployed | 862 | 23.14 |
| Alcohol dependence | 189 | 5.07 |
| Drug dependence | 215 | 5.77 |
| Psychosis (PSQ 3+) | 143 | 3.84 |
| Anxiety disorder | 116 | 3.11 |
| Depressive disorder | 128 | 3.44 |
| STD | 472 | 12.67 |
| Paid sex worker (ever) | 355 | 9.53 |
| Anal sex | 507 | 13.61 |
| Coercive sex | 675 | 18.12 |
| > 10 sexual partners (last year) | 622 | 16.70 |
| Rare contraceptive use | 744 | 19.97 |
| Sexually active MSM | 421 | 11.30 |
| Repeated violence | 197 | 5.29 |
| IPV | 136 | 3.65 |
| Fear of violent victimisation | 367 | 9.85 |
| Carried a weapon | 293 | 7.87 |
| Gang membership | 513 | 13.77 |
| Peers encourage crime | 311 | 8.35 |
| Ever in prison | 10 | 0.27 |

*Supplementary Figure 1. Proportion of responders to health, violence and criminal outcomes, by location (survey) and ethnic group*


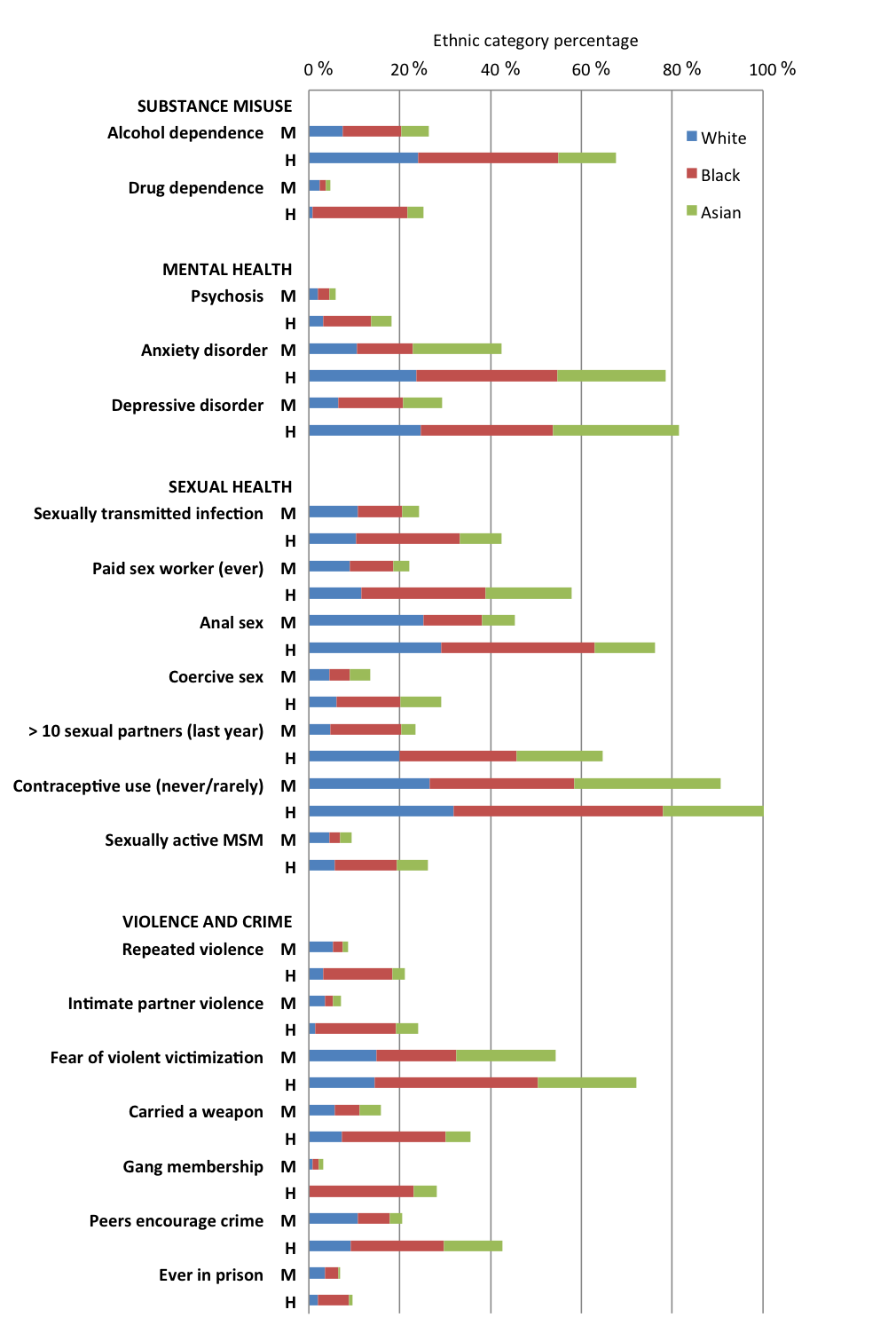


**Note**. M Main survey, H Hackney

***Supplementary Figure 2.*** *Confirmatory factor model for mental health, substance misuse, violence/crime and sexual health factors,*

*and a second-order general syndemic factor model*


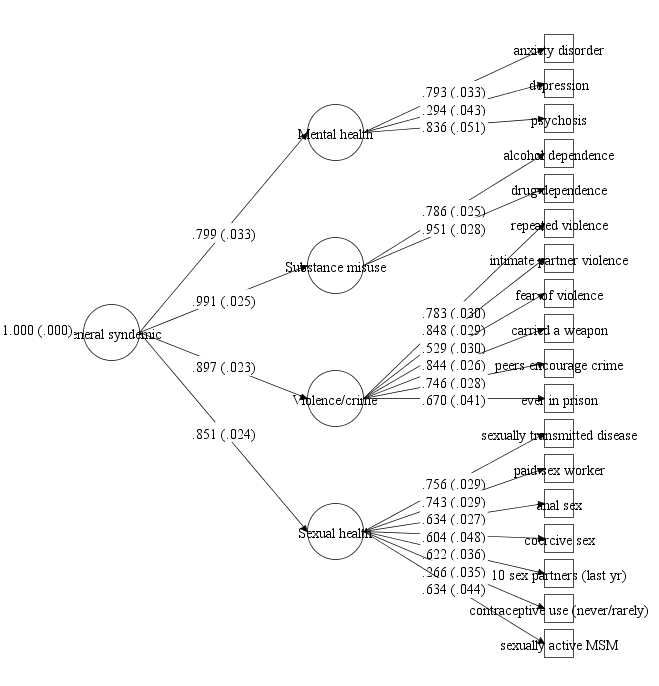


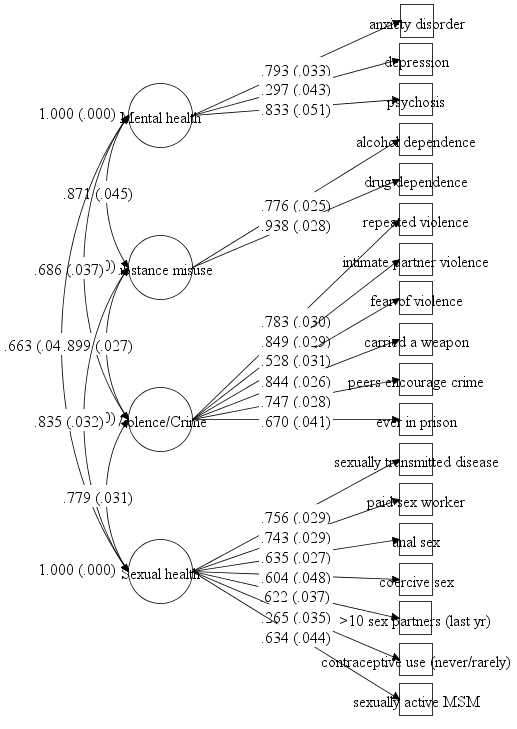

Supplement: Supplementary file 1 [file S0007125019002034sup001.docx]
